# Development of an HDR‐BT QA tool for source position verification

**DOI:** 10.1002/acm2.13063

**Published:** 2020-11-02

**Authors:** Yu Kumazaki, Ryuta Hirai, Mitsunobu Igari, Nao Kobayashi, Shohei Okazaki, Takanori Abe, Tomoaki Tamaki, Shin‐ei Noda, Shingo Kato

**Affiliations:** ^1^ Department of Radiation Oncology Saitama Medical University International Medical Center Saitama Japan; ^2^ Department of Radiation Oncology Gunma University Graduate School of Medicine Maebashi Gunma Japan; ^3^ Department of Radiation Oncology Fukushima Medical University Fukushima Japan

**Keywords:** applicator modeling, applicator offset, HDR, QA phantom, source position

## Abstract

**Purpose:**

This study aimed to develop a high‐dose‐rate brachytherapy (HDR‐BT) quality assurance (QA) tool for verification of source positions, and to report on its effectiveness.

**Methods:**

We fabricated a cuboid phantom measuring 30 × 30×3 cm^3^ with spaces to embed Fletcher‐Williamson tandem and ovoid applicators. Lead‐based, cylindrically shaped radiopaque markers, which scatter radiation and blacken the Gafchromic^®^ RTQA2 films placed on the applicators, were inserted into the phantom to determine the applicator tip and reference source positions. A three‐dimensional image‐guided brachytherapy (3D‐IGBT) plan was generated, and the source positions on the film and radiation treatment planning system (RTPS) were verified with the tool. Source position errors were evaluated as the distance in the applicator axis direction between the source position and the center position of two radiopaque marker pairs.

**Results:**

Source position errors on the film and RTPS were in good agreement with one another and were all within 0.5 mm for all applicators. Offset values of each applicator were in good agreement with the value determined in treatment planning (6 mm). The expanded measurement uncertainty of our QA tool was estimated to be 0.87 mm, with a coverage factor k of 2.

**Conclusions:**

Our new HDR‐BT QA tool developed for comprehensive source position verification will be useful for cross checking actual source positions and planned source positions on the RTPS.

## 1 INTRODUCTION

Brachytherapy is a radiotherapy in which small encapsulated radioactive sources are placed within or in close proximity to a target volume.^1^ High‐dose‐rate brachytherapy (HDR‐BT) using ^192^Ir sources has been performed for various types of tumors, including uterine cervix, prostate, breast, head and neck, skin, bronchial, and esophageal tumors.^2^, ^3^, ^4^, ^5^, ^6^, ^7^, ^8^ Since radioactive sources are placed near the tumor, a very high radiation dose can be delivered to the tumor site, while minimizing doses to surrounding normal tissues due to the rapid fall‐off in the dose distributions according to the inverse square law. Three‐dimensional (3D) image‐guided brachytherapy (3D‐IGBT) using 3D images, such as computed tomography (CT) or magnetic resonance (MR) images, has recently become a standard,^9^, ^10^ although traditional x‐ray‐based two‐dimensional (2D) treatment planning is still used. In 2D‐BT, markers of x‐ray catheters are used to determine the first source position in treatment planning. In 3D‐IGBT, the first source position is determined either by using x‐ray catheters in CT image sets or line makers in MR image sets, or based on the applicator offset, that is, the distance from the applicator tip to the first dwell position. This is a very important point, as each source position is determined relative to the first source position. However, the value of applicator offset, which is not always provided in datasheet of manufacture, needs be confirmed by users themselves during commissioning, because if the value on the radiation treatment planning system (RTPS) is inaccurate, the treatment plan cannot be accurately implemented and severe radiation complications can occur. Since errors related to applicator offset cannot be detected by mechanical testing alone (e.g., verification of dummy source positions using radiographs), verification of active source positions, such as the end‐to‐end test, must also be performed. Combining autoradiography and radiography in the same radiographic or radiochromic film is not considered an end‐to‐end test, as this method does not allow for cross checking of actual source positions and planned source positions on the RTPS. Thus, there is a need to develop an HDR‐BT quality assurance (QA) tool to verify active source positions.

Several studies have reported methods for HDR‐BT source position verification.^11^, ^12^, ^13^, ^14^, ^15^, ^16^, ^17^ Radiochromic dosimetry films have been used to verify active source positions for HDR‐BT,^11^ although a lack of information on reference positions makes it difficult to quantitatively assess source positions. To address this issue, some have suggested methods to add information on films, such as by superimposing a source autoradiograph on a radiograph of dummy sources inside the applicator,^12^ manually marking reference positions,^13^ or utilizing a tungsten wire.^14^ Meanwhile, others have reported on methods to obtain source information without using radiographs, for example, with a plastic scintillator block and CCD camera^15^ or a transparent applicator.^16^ However, none of these methods involved the use of a comprehensive verification system to cross check the actual source positions and planned source positions on the RTPS. Recently, Okamoto et al. developed a comprehensive source position verification system^17^; however, no information regarding applicator offset was provided. Moreover, no credentialing exists for source positions in brachytherapy, and no commissioning tool to perform comprehensive source position testing is commercially available. While commercially available autoradiography devices are useful test tools for mechanical source positioning, the applicator reconstruction process is not included. Therefore, this study aimed to develop an HDR‐BT QA tool that allows for verification of actual source positions and planned source positions on the RTPS.

## 2 MATERIALS AND METHODS

### 2.A Development of an HDR‐BT QA tool

**Fig. 1:**
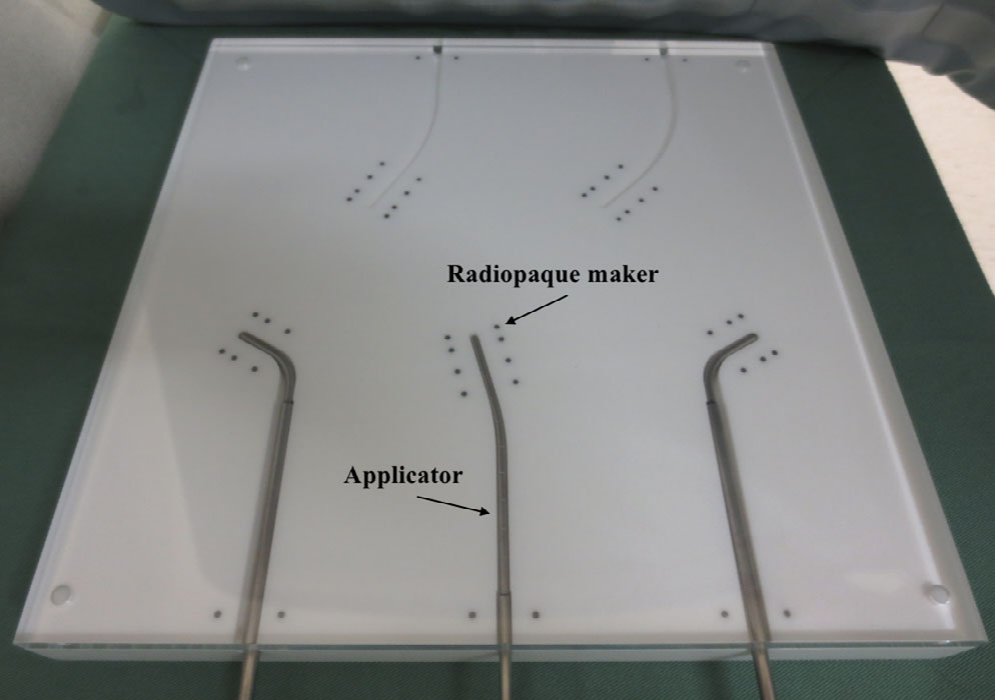
HDR‐BT QA phantom for source position verification with Oncentra^®^ applicator modeling.

**Fig. 2:**
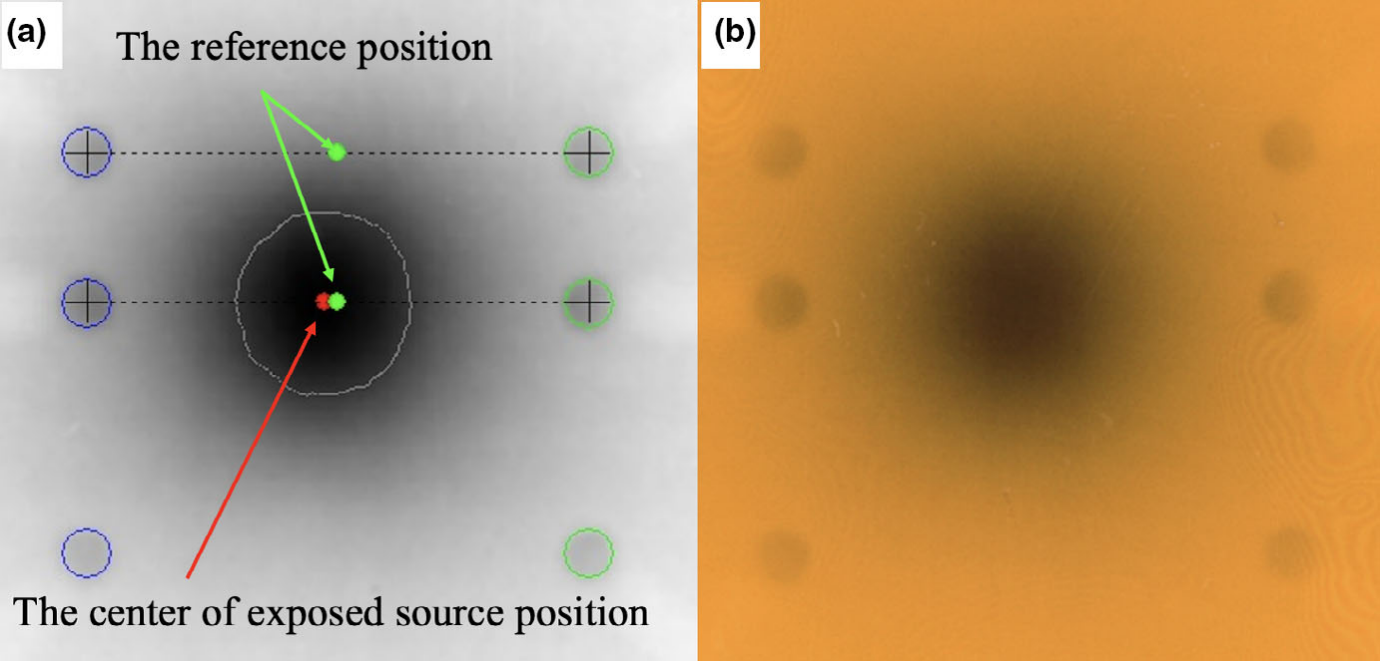
(a) Automated analysis software for source position verification with Oncentra^®^ applicator modeling, (b) RTQA2 Gafchromic® film exposed by Ir‐192.

**Fig. 3:**
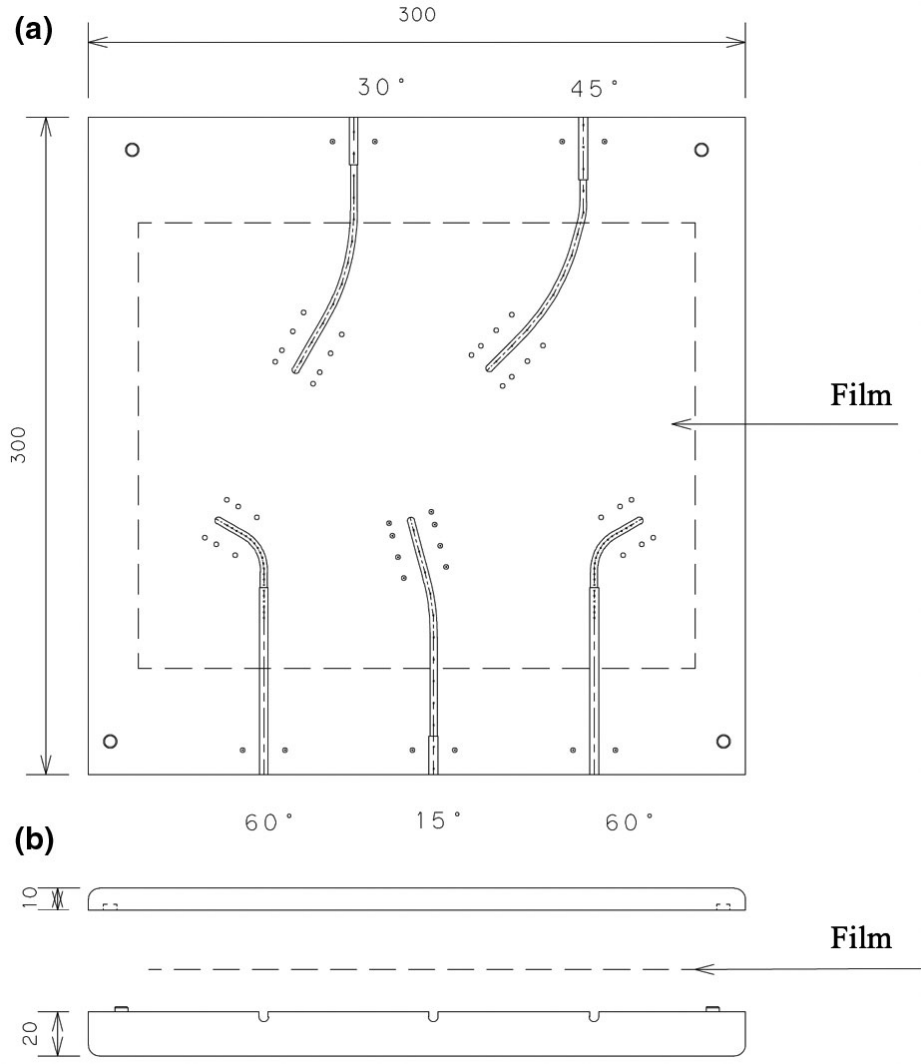
Position of the film in the phantom. (a) Anterior–posterior view, (b) inferior–superior view.

We developed an HDR‐BT QA phantom (Fig. 1) and automated analysis software (Fig. 2) for verification of source positions with Fletcher‐Williamson applicators (Elekta Oncology Systems, Crawley, UK). We fabricated a cuboid phantom measuring 30 × 30 × 3 cm^3^ made of Acrylonitrile‐Butadiene‐Styrene (ABS) plastic 1.05 g/cm^3^ in density. The phantom has spaces to embed tandem applicators at three angles (15, 30, and 45 degrees) as well as ovoid applicators. Lead‐based, cylindrically shaped radiopaque markers 11.34 g/cm^3^ in density, measuring 2 mm in diameter and 2.5 mm in height, were inserted into the phantom to determine the applicator tip and reference source positions. Six markers were placed bilaterally 10 mm away from the applicator in the short axis, and 0, 6, and 16 mm caudal from the tip of the applicator in the long axis (Fig. 1). These markers are designed to scatter radiation and blacken Gafchromic^®^ RTQA2 films (Ashland Specialty Ingredients, New Jersey, USA) placed on the applicators (Figs. 2, 3).

### 2.B Source position measurement workflow

**Fig. 4:**
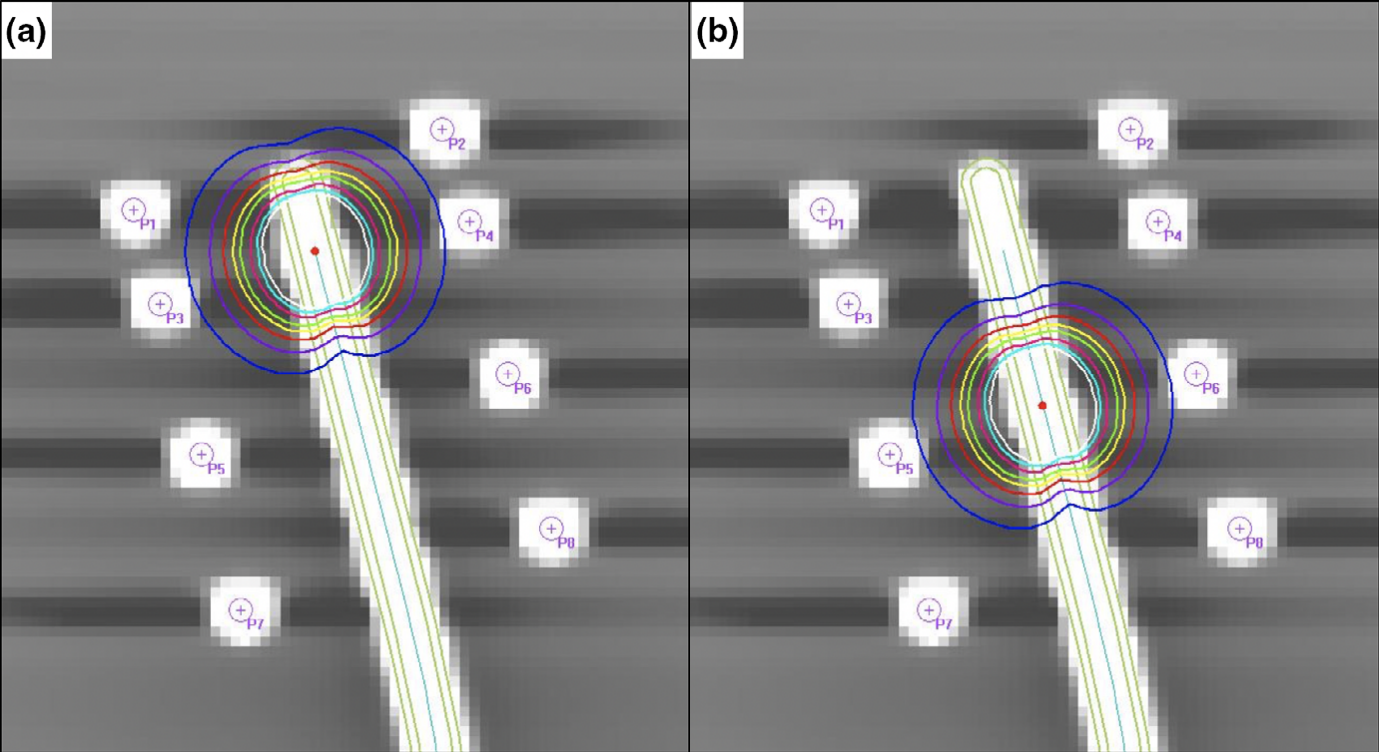
Examples of treatment planning using applicator modeling for the Fletcher Williamson Asia Pacific applicator in the Oncentra^®^ treatment planning system. (a) The source was placed 6 mm from the applicator tips. (b) The source was placed 10 mm from the first source position.

The phantom was scanned by an x‐ray CT scanner (Aquilion LB, Canon Medical Systems Corp., Tochigi, Japan) with a slice thickness of 1 mm. CT images were imported to the RTPS (Oncentra® Brachy, Elekta Oncology Systems, Crawley, UK), and 3D‐IGBT plans were generated (Fig. 4). Applicator modeling was used to reconstruct source pathways. The first source was positioned 6 mm caudal from the applicator tip, as the value of applicator offset was determined to be 6 mm in a film‐based mechanical source positioning test performed prior to this study. The second source was positioned 10 mm caudal from the first source. The dwell time was set at about 10 s to produce a radiograph of adequate density on Gafchromic^®^ RTQA2 films. Each plan was generated separately so as not to affect the determination of each exposed position on the film. The plan was exported to the Treatment Control System (TCS), the console of the remote afterloading system. The MicroSelectron‐v2r source, an ^192^Ir core measuring 3.5 mm in length and 0.6 mm in diameter, enclosed in a stainless steel capsule measuring 4.5 mm in length and 0.9 mm in diameter, was used (Elekta Oncology Systems, Crawley, UK) ^18^. ^192^Ir source activity was 362 GBq. After each applicator was connected via transfer tube to the HDR afterloader, films were exposed in accordance with the plans. In addition, we created reference points at the center of each radiopaque marker on the RTPS to evaluate source positions and applicator offset in treatment planning (Fig. 4).

### 2.C Evaluation of source position errors

After radiation exposure, exposed films were scanned with the EPSON ES‐10000G flatbed scanner (Seiko Epson Corp., Nagano, Japan) at 300 dpi resolution. Source position error on the film (${\mathrm{SPE}}_{\mathrm{film}}$) was evaluated as the distance in the applicator axis direction between the exposed source position and the reference position (i.e., the center position of the two radiopaque marker pairs) using in‐house software. Positive error values indicate that the source was positioned cephalad to the applicator tip (Fig. 2). The center of the exposed source position was determined as the center of the 80% isodose level on the film, since it was clearly distinguishable from the radiopaque marker position. The center position of each radiopaque marker was determined as the center of the outline of the marker using a differential filter, since the dose profile at the maker was not symmetrical. The positions of the source and radiopaque markers on the RTPS were obtained from the Digital Imaging and Communications in Medicine Radiation Therapy (DICOM‐RT) plan using in‐house software. Source position error on the RTPS (${\mathrm{SPE}}_{\mathrm{RTPS}}$) was evaluated in the same way as ${\mathrm{SPE}}_{\mathrm{film}}$. Total source position error (${\mathrm{SPE}}_{\mathrm{total}}$) was evaluated as the difference between ${\mathrm{SPE}}_{\mathrm{film}}$ and ${\mathrm{SPE}}_{\mathrm{RTPS}}$. Finally, applicator offset was evaluated as the distance in the applicator axis direction between the reference position at the applicator tip and the first source position.

## 3 RESULTS

End‐to‐end measurements of tandem and ovoid applicators were performed five times each. ${\mathrm{SPE}}_{\mathrm{film}}$ and ${\mathrm{SPE}}_{\mathrm{RTPS}}$ were within 0.5 mm for all applicators (Table 1). The first and second exposed source positions on the film were in good agreement with those of the RTPS for all applicators (Table 1). The values of applicator offset obtained for each applicator were in good agreement with the value determined in treatment planning (i.e., 6 mm) (Table 1).

**Table 1:** Summary of source position errors (SPE) and applicator offset on the film and the RTPS. Data are shown in mean ± SD.

	Tandem			Ovoid
	15 deg.	30 deg.	45 deg.	
Applicator offset on the RTPS (mm)	6.0±0.1	6.2±0.2	5.9±0.1	6.1±0.2
Applicator offset on the film (mm)	5.8±0.1	6.2±0.1	6.2±0.2	6.0±0.2
$SPE_{\mathrm{RTPS}}$ at 1st source position (mm)	0.1±0.2	0.0±0.2	0.0±0.2	0.1±0.2
$SPE_{\mathrm{film}}$ at 1st source position (mm)	0.1±0.0	−0.2±0.0	−0.3±0.2	0.0±0.2
$SPE_{\mathrm{RTPS}}$ at 2nd source position (mm)	0.1±0.1	0.1±0.2	0.0±0.1	0.1±0.2
$SPE_{\mathrm{film}}$ at 2nd source position (mm)	0.0±0.1	−0.2±0.1	−0.3±0.2	−0.2±0.2
$SPE_{\mathrm{total}}$ at 1st source position (mm)	0.0±0.2	−0.2±0.2	−0.3±0.4	−0.1±0.4
$SPE_{\mathrm{total}}$ at 2nd source position (mm)	−0.1±0.2	−0.3±0.4	−0.3±0.1	−0.3±0.2

Measurement uncertainties of our QA tool were categorized according to the “Guide to the expression of uncertainty in measurement (GUM)”.^19^ They included manufacturing errors in the position of radiopaque markers, as well as uncertainty in the determination of the radiopaque marker position center and the source position center on the film and RTPS (Table 2). Uncertainties regarding the transfer tube length and source position were not categorized in the present study, since they were evaluated as measurement errors. The phantom developed in this study was made to order, and manufacturing errors in the position of the radiopaque markers were estimated to be 0.1 mm, although nominal accuracy was <0.1 mm. The uncertainty of the radiopaque marker position center and the source position center on the film was estimated to be 0.08 mm based on the pixel resolution of images scanned at 300 dpi; these positions were automatically obtained using in‐house software. The uncertainty of the radiopaque marker position center and the source position center on the RTPS was estimated to be 0.29 mm based on the pixel resolution of CT images in the coronal plane at 1‐mm slice thickness. Although the source position center on the RTPS depended on the position of the applicator tip in the reconstructed source pathway, it also depended on CT slice thickness. We also confirmed that changes in the center of the exposed source position were within 0.1 mm, even when 70‐90% isodose levels were used to calculate the exposed source position center. Thus, the expanded measurement uncertainty was estimated to be 0.87 mm with a coverage factor k of 2.

**Table 2:** Uncertainties of the HDR‐BT QA tool.

	Uncertainty (mm)
Manufacturing errors in the position of radiopaque markers	0.10
Determination of the radiopaque marker position center on the film	0.08
Determination of the exposed source position center on the film	0.08
Determination of the radiopaque marker position center on the RTPS	0.29
Determination of the dwell position center on the RTPS	0.29
Combined standard uncertainty	0.44
Expanded uncertainty (k =2)	0.87

Symbol k represents the coverage factor.

All uncertainties were estimated as type B.

RTPS: Radiation treatment planning system.

## 4 DISCUSSION

We developed an HDR‐BT QA tool for source position verification, which allows for quantitative verification of actual source positions and planned source positions on the RTPS, as well as evaluation of source position errors. All source position errors measured in the present study were within 1 mm (Table 1). These results satisfied the tolerance for source positional accuracy specified by the AAPM Task Group 56,^1^ suggesting that our QA tool can be useful as a commissioning tool to perform a comprehensive source position test. To the best of our knowledge, no report has described HDR‐BT QA tools having these functions.^11^, ^12^, ^13^, ^14^, ^15^, ^16^

**Fig. 5:**
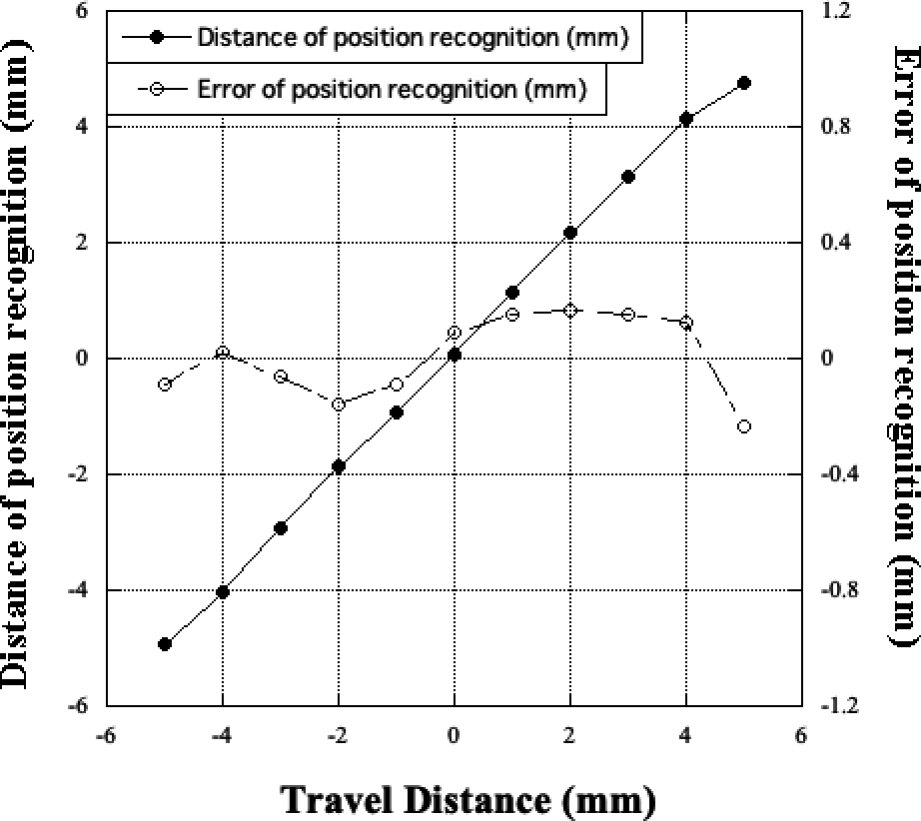
Position recognition accuracy of the HDR‐BT QA tool.

Our QA tool allows for verification of actual source positions, as the interaction between Ir‐192 gamma ray and radiopaque markers blackens radiographic films (Fig. 2). Thus, there is no need to use an x‐ray machine to obtain information on the dummy source position or manually mark reference points to determine the exposed source positions. A previous study reported on source position verification using an Ir‐192 source itself.^14^ In that study, tungsten wires were placed to obtain information on the reference position so that the exposed source positions could be determined on the film. However, this method allowed only for evaluation of source position errors in the region where tungsten wires were placed, and the shadings of the tungsten wires generally appeared to be unclear. In contrast, our QA tool allowed for evaluation of source position errors whenever the sources were placed between the radiopaque markers. Figure 5 shows the position recognition accuracy of our tool when the exposed source position was moved ±5 mm from the reference position. These results suggest that our QA tool can accurately evaluate source positions, even when the exposed source positions are located between radiopaque markers.

In this study, we evaluated source position errors in the applicator axis direction, but source position errors perpendicular to the applicator axis were not evaluated. Although the difference from the applicator center was within 1 mm (Table 3), these errors might have influenced the dose distributions. A future study will be needed to address this point.

**Table 3:** Source position errors (mm) perpendicular to the applicator axis on the film. Data are shown in mean ± SD.

	Tandem			Ovoid
	15 deg.	30 deg.	45 deg.	
1st source position (mm)	0.7±0.1	0.6±0.1	0.4±0.1	0.4±0.1
2nd source position (mm)	0.5±0.1	0.5±0.1	0.6±0.1	0.3±0.1

Our QA tool had an expanded measurement uncertainty (k = 2) within 0.87 mm (Table 2). This value was lower than that of a previously reported method.^17^ The expanded measurement uncertainty (k = 2) of the tool, except for the uncertainty in the RTPS, was within 0.3 mm, which was lower than that of another reported method.^13^ One of the reasons may have been the uncertainty of the radiopaque marker position center.

There are several limitations in this study. First, our phantom was designed to confirm source positions with Fletcher‐Williamson applicators used in the MicroSelectron HDR system. Therefore, it was not possible to verify source position errors with other applicators. However, since the phantom can be modified for different types of applicators, evaluation of source positions as well as the offset value for other applicators will be possible. Second, we cannot verify source strength, which is also important in QA of HDR‐BT, as our HDR‐BT QA tool is specifically designed for source position verification.

## 5 CONCLUSION

A new HDR‐BT QA tool was developed for source position verification and confirmation of applicator offset. This tool will be useful not only for cross checking actual source positions and planned source positions on the RTPS, but also as a postal audit tool given its low measurement uncertainty and simple methodology.

## CONFLICT OF INTEREST

The authors declare no conflict of interest associated with this manuscript.
